# Structural and Functional Consequences Induced by Post-Translational Modifications in **α**-Defensins

**DOI:** 10.1155/2011/594723

**Published:** 2011-08-28

**Authors:** Enrico Balducci, Alessio Bonucci, Monica Picchianti, Rebecca Pogni, Eleonora Talluri

**Affiliations:** ^1^School of Biosciences and Biotechnologies, University of Camerino, Gentile III da Varano Street, 62032 Camerino, Italy; ^2^Department of Chemistry, University of Siena, 53100 Siena, Italy; ^3^Research Center, Novartis Vaccines and Diagnostics, Fiorentina 1 Street, 53100 Siena, Italy; ^4^Department of Evolutionary Biology, University of Siena, 53100 Siena, Italy

## Abstract

HNP-1 is an antimicrobial peptide that undergoes proteolytic cleavage to become a mature peptide. This process represents the mechanism commonly used by the cells to obtain a fully active antimicrobial peptide. In addition, it has been recently described that HNP-1 is recognized as substrate by the arginine-specific ADP-ribosyltransferase-1. Arginine-specific mono-ADP-ribosylation is an enzyme-catalyzed post-translational modification in which NAD^+^ serves as donor of the ADP-ribose moiety, which is transferred to the guanidino group of arginines in target proteins. While the arginine carries one positive charge, the ADP-ribose is negatively charged at the phosphate moieties at physiological pH. Therefore, the attachment of one or more ADP-ribose units results in a marked change of cationicity. ADP-ribosylation of HNP-1 drastically reduces its cytotoxic and antibacterial activities. While the chemotactic activity of HNP-1 remains unaltered, its ability to induce interleukin-8 production is enhanced. The arginine 14 of HNP-1 modified by the ADP-ribose is in some cases processed into ornithine, perhaps representing a different modality in the regulation of HNP-1 activities.

## 1. Introduction

Antimicrobial host defense peptides (AMPs) are widely distributed throughout all kingdoms of life and represent one of the most ancient host defense factors. All of them display microbicidal activity against a wide spectrum of Gram-negative and Gram-positive bacteria, fungi, and viruses [1]. The two main classes of AMPs produced by humans are represented by defensins and by the single cathelicidin LL-37, a peptide of 37 amino acids beginning with two leucin residues (L-L). Moreover, humans produce basic histatins, which are released in the oral cavity [2], dermicidins from the skin, and granulysin, which are released by proteolytic processing in sweat, and in leucocytes, respectively [3, 4]. Defensins are cationic multifunctional arginine-rich peptides with molecular masses ranging from 3.5 to 6 kDa, characterized by three intramolecular disulfide bonds. Based on the connectivity of the six conserved cysteine residues and sequence homology, human defensins are grouped into *α*- and *β*-defensins. Human neutrophil peptides, HNP-1 to 4, are the major component of the azurophilic granules of neutrophils while the *β*-defensins are mainly expressed in epithelial cells of various tissues and organs [5–7]. HNP-1 is synthesized as prepropeptide, which is processed via different sequential proteolytical cleavage steps to the final functional peptide of 30 amino-acid. Cleavage is by far the most studied post-translational modification for AMPs and is also the topic of several excellent reviews [1, 8, 9]. In this review we summarize the current knowledge regarding the influence of ADP-ribosylation on both structure and function of peptides belonging to the innate immune system and we describe this post-translational modification at the molecular level. Moreover, we aim to describe the strong impact that this modification exerts on the ability of HNP-1 to kill bacteria but also on the cytotoxicity versus eukaryotic cells. ADP-ribosylated-HNP-1 are present in the airways of patients with inflammatory-based diseases [10], therefore this novel discovery has the potential to open up new avenues of investigation with regard to pulmonary inflammatory diseases, which can be translated into tools for therapeutic applications. 

## 2. Biochemical Characterization of Mono-ADP-Ribosylation

**Figure 1:**
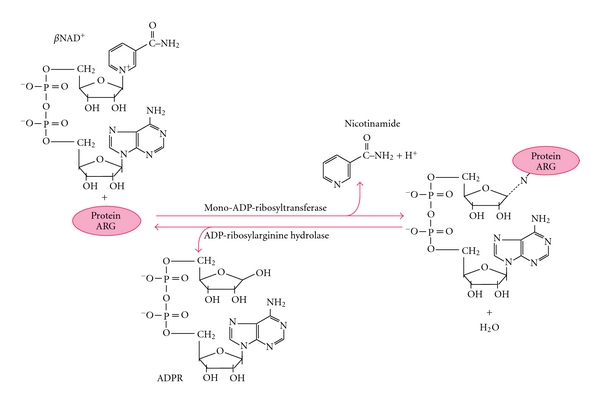
Schematic representation of the enzyme-catalyzed reversible mono-ADP-ribosylation reaction. The diagram shows the enzymatic transfer of the ADP-ribose portion from NAD to the guanidine group of an arginine target. Nicotinamide, the remaining portion of NAD, is simultaneously released. The native arginine is restored by the reaction catalyzed by an ADP-ribosylarginine hydrolase that cleaves the *α*-glycosidic bond with the release of ADP-ribose.

Mono ADP-ribosylation is a reversible enzymatic post-translational modification, which alters the chemical and functional properties of the modified proteins [11]. In this reaction mono-ADP-ribosyltransferases (ARTs) catalyze the covalent attachment of an ADP-ribose unit from NAD^+^ to specific amino-acid residues with the simultaneous release of nicotinamide (Figure 1). The ART activity has been initially described in prokaryotes as one of the main mechanism of bacterial pathogenesis [12–14]. More recently five enzymes (ART1, ART2, ART3, ART4, and ART5) related to mono-ADP-ribosylating toxins have been identified in eukaryotes. Among them only ART1, ART2, and ART5 contain the R-S-EXE triad signature typical of a subfamily of arginine-specific ARTs. These enzymes selectively recognize and modify Arg as amino-acid acceptor. This signature is absent in the other two mammalian transfer enzymes ART3 and ART4, whose activity has not been characterized yet. ARTs are glycosylphosphatidylinositol (GPI) linked to the membrane with the catalytic site at the outer surface of cells with the exception of ART5, which represents a secretory form of ART [15, 16]. ADP-ribosylation of arginine residues can influence the function of target proteins by several mechanisms. The ADP-ribosylation reaction introduces a large and bulky group on the target protein, which is fivefold bigger (540 Da) than the average size of the amino-acid. Therefore, the presence of a single or multiple ADP-ribose modifications can considerably influence the structure and the size of antimicrobial peptides with an MW ranging between 3 and 6 kDa. The addition of ADP-ribose can induce a conformational change in the target protein, which often results in an activating effect or in the creation of a new docking site for ADP-ribose binding domains in other proteins [17]. At the same time the presence of ADP-ribose can also impede the sterical interaction with molecular partners. In addition, at neutral pH while the arginine carries one positive charge, the ADP-ribose is negatively charged at the phosphate moieties. Therefore, the transfer of one or more negatively charged ADP-ribose moieties has the potential to considerably reduce the positive charge of the modified Arg and overall the cationicity of antimicrobials. 

## 3. Physiological Implications of Post-Translational Modifications of HNP-1

**Figure 2:**
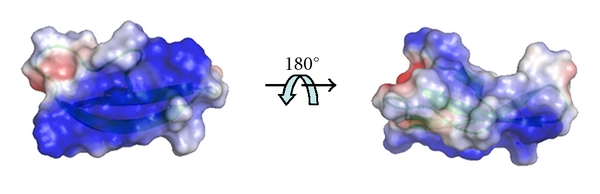
Density electrostatic surface map of HNP-1 peptide. Positive charges conferred by arginine residues are shown in blue; the negative charge conferred by glutamic acid residue is in red. The secondary structure of the peptide was taken from PDB database (3GNY) and the relative density electrostatic surface map was obtained using *PyMOLWin* software.

**Figure 3:**
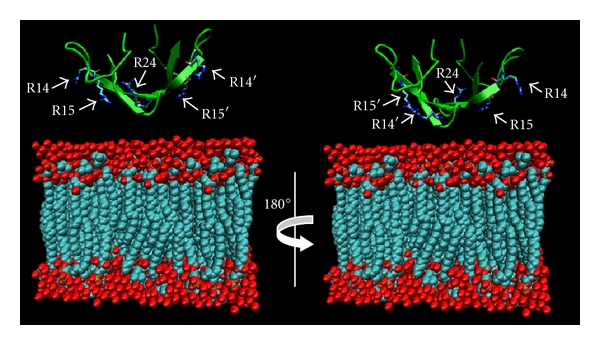
Schematic structure of the dimeric form of HNP-1 interacting with the cell membrane. Arg residues that confer a positive net charge to HNP-1 (green) and that are involved to membrane interaction are highlighted in cyan and blue. Negatively charged groups of the cell membrane such as phosphatidyl glycerol or cardiolipin head groups are highlighted in red. Arg14 and Arg24 is the primary and secondary site of ADP-ribosylation, respectively. The schematic representation was made using *Visual Molecular Dynamics* (VMD) software; HNP-1 secondary structure was taken from PDB database (3GNY) while cell membrane model was created with VMD tool.

**Figure 4:**
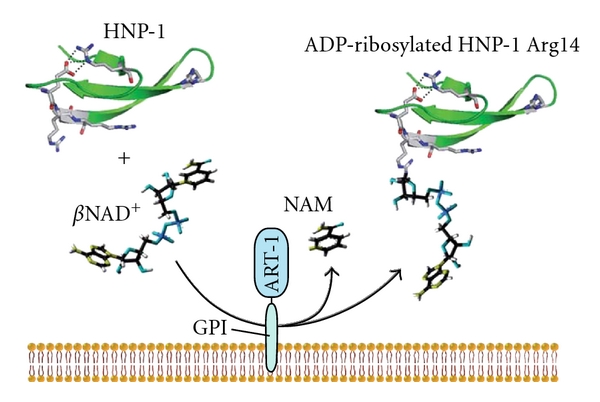
Schematic diagram of the HNP-1 ADP-ribosylation. The picture shows that ART1 catalyzes the transfer of an ADP-ribose unit from NAD to HNP-1 on Arg14 with simultaneous release of nicotinamide (NAM) in the extracellular space. ART1 is anchored via a GPI tail to the outer leaflet of the cell membrane.

The localization of ART1 on the surface of ciliated and intermediate epithelial cells lining the airways [18] led to the proposal that potential substrates for ART1-specific ADP-ribosylation could be present in the extracellular milieu. As shown in Table 1, *α*- and *β*-defensins are cationic peptides with a high content of Arg residues which are the amino-acid target for the ART1 catalyzed ADP-ribosylation. Azurophilic granules in inflammatory cells mainly store HNP-1 molecules. Since their main function is to defend the lung against pathogenic microorganisms, they are secreted at high concentration at the site of inflammation in the airways by neutrophils [19]; therefore, they could represent an ART1 substrate. In aqueous solution, HNP-1 is arranged in a dimeric form, where each monomer presents a positive net charge equal to +3 (Figure 2), conferred by four arginine residues (Arg5, Arg14, Arg15, and Arg24) and a negative glutamic acid (Glu13). The last three arginine residues of HNP-1 are directly involved in membrane perturbation while Arg5 is involved in the formation of a salt bridge with Glu13. The positively charged arginine residues provide an effective means of attracting dimeric HNP-1 peptide to target membranes facilitating its interaction with negatively charged surfaces, such as phosphatidyl glycerol or cardiolipin head groups (Figure 3). Previous studies demonstrate that the replacement of Arg residues by neutral or oppositely charged amino acids, and modification of the amino-acid sequence leads to dramatic reduction or loss of activity in bacterial killing not only in HNP-1 peptide but also in other cationic antimicrobial peptides [20–22]. It has been shown (Figure 4) that HNP-1 is ADP-ribosylated when assayed *in vitro* in the presence of ART1 and NAD^+^ [23]. The primary site of modification is Arg14, but also Arg24 is shown to be ADP-ribosylated after a longer incubation time. Interestingly, Arg14 and Arg24, as described previously, are directly involved in membrane interaction. Arg5, which is involved in a salt bridge, is not targeted by ART1. Both forms were isolated from bronchoalveolar lavage (BAL) of volunteer with history of smoking [10]. The ADP-ribosylation of selected Arg might well correlate with recent discoveries, which show that antibacterial activity is strictly dependent by cationicity [24] and that only selective Arg support this activity [25]. While ART2 slightly modifies HNP-1, ART5 is completely inactive on HNP-1 [10] supporting the evidence of a specific modification since the mouse ART1 ortholog and ART1 GPI-linked to the skeletal muscle membrane modifies HNP-1 with the same intensity [10]. The ADP-ribosylated-HNP-1 exhibited a strong reduction of antimicrobial activity compared to the unmodified peptide. Moreover, while the chemotactic activity of the modified and unmodified HNP-1 resulted unaltered, the ability of HNP-1 to release interleukin-8 (IL-8) from A549 cells was enhanced by the presence of ADP-ribose linked to Arg14 [23]. This is in line with the idea that the ADP-ribosylated-HNP-1 is a completely different molecule, which possesses unique biological characteristics that influence the ability to recruit neutrophils in the site of inflammation through the release of IL-8 from epithelial cells. This latter is an important point since mono- and di-ADP-ribosylated forms of HNP-1 were found in BAL of patients with pulmonary diseases such as idiopathic pulmonary fibrosis and asthma. Though the neutrophils in these diseases were recruited in high number, and HNP-1 and other isoforms were found in their granules, modified HNP-1 were not present. This suggests that HNP-1 have to be released from neutrophils to be ADP-ribosylated by ART1, which is expressed at the outer surface of epithelial cells. High concentrations of HNP-1 exert cytotoxic effects towards epithelial cells. In airways of patients with pulmonary diseases the concentration of HNP-1 produced by epithelial cells and infiltrating neutrophils, is high. Therefore, these cytotoxic effects may well be physiologically relevant at sites of inflammation and could result in a high local concentration of HNP-1, thus leading to cytotoxicity. Since electrostatic interactions play a key role in the interaction with biological membranes, it is conceivable that modifications like ADP-ribosylation, which link a negatively charged ADP-ribose moiety to the Arg14 and Arg24, may reduce the interaction with biological membranes and consequently the cytotoxicity that HNP-1 exerts when its concentration is high. Interestingly, the cytotoxicity of HNP-1 towards epithelial cells is drastically reduced when the peptide is ADP-ribosylated [23]. 

## 4. Ornithinylation and Phosphorylationof HNP-1

**Figure 5:**
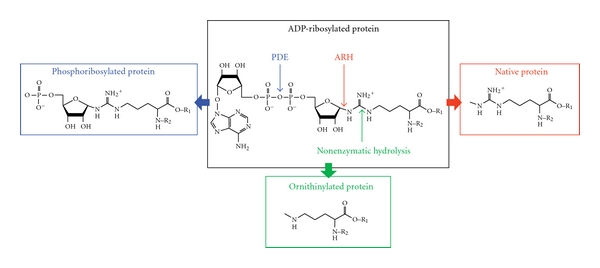
Different mechanisms of processing and cleavage of ADP-ribosylated proteins. The complete removal of the ADP-ribose, catalyzed by an ADP-ribosylarginine hydrolase is indicated in red. The picture in blue frame shows the phosphodiesterase-catalyzed cleavage of the ADP-ribosylated-protein, giving a phosphoribosylated protein. The picture in the green frame represents the nonenzymatic processing of the ADP-ribosylated protein to the ornithinylated form as described for HNP-1.

Post-translational modifications of HNP-1 are not restricted to the ADP-ribosylation. As shown in Table 1 defensins are also rich in Tyr, which are the sites, phosphorylated by tyrosine kinases. Using a novel phosphoproteomic approach to identify and validate phosphotyrosine sites it has been shown that the Tyr21 (YGTCIY*QGR) in HNP-1 and HNP-3 is phosphorylated in cancer cell lines [26]. Because these studies represent an early step in the characterization of the tyrosine kinase signaling in lung cancer, the role that this modification plays in the defensin activities has to be established. The phosphoryl group is only 80 Da with respect to ADP-ribose, but it is interesting to note that in model peptide substrate phosphorylation affects ADP-ribosylation in the nearby arginyl residues [27]. This possible link between phosphorylation and ADP-ribosylation could be relevant in the context of HNP-1 in which the Arg14 and the Arg24 are close to the Tyr21. Another modification that seems to occur *in vivo* is the generation of the ornithyl-HNP-1 from ADP-ribosylated-HNP-1 [28]. The guanidino carbon of the ADP-ribosylated-arginine can undergo nonenzymatic hydrolysis resulting in the replacement of the ADP-ribosylarginine by the noncoded amino-acid ornithine (Figure 5). This modification is specific, since it targets only the ADP-ribosylated-arginine at position 14. Moreover, it prevents the modified Arg to serve as substrate for subsequent ADP-ribosylation since ornithine is not recognized as substrate by ART1. BAL of patients with idiopathic pulmonary fibrosis and asthma other than mono- and di-ADP-ribosylated-HNP-1 contained HNP-1 ADP-ribosylated at Arg24 with ornithine replacing Arg in position 14 suggesting a relevance of this conversion for the innate immunity in the airway [28]. Interestingly, when 3 of the 4 Arg (not Arg5) were replaced by ornithine the bactericidal activity was decreased suggesting that the conversion of arginine with ornithine can be important in altering HNP-1 activities [24]. Moreover, the chemical degradation of the ADP-ribosylated-arginine in ornithine creates a secondary post-translational modification that could potentially amplify the ability of the cell to regulate the biological activities of the HNP-1 and consequently the immune response in the airways. 

## 5. Processing and Reversibility of the ADP-Ribose Moiety

Like many post-translational modifications with regulatory functions, mono-ADP-ribosylation of Arg is a potentially reversible process that could lead to a complete or partial removal of the ADP-ribose unit. A full removal of the ADP-ribose unit from the modified amino-acid is catalyzed by a specific mammal ADP-ribosylarginine hydrolase-1 (ARH1) [29], which regenerates free Arg for a new ADP-ribosylation cycle (Figure 5). Since ARH1 is localized in the intracellular compartment and modification of HNP-1 is catalyzed by the extracellular ART1, the restoration of unmodified HNP-1 due to the action of ARH1 is unlikely. In fact at least one of the two proteins should translocate across the membrane. Partial reversal of the ADP-ribose moiety may result from the action of extracellular phosphodiesterases that cleave adenosine-5′-monophopshate from ADP-ribose yielding a ribosyl moiety attached to the target Arg [30]. This modification precludes a new ADP-ribosylation of the target Arg since it leaves attached a phosphoribose molecule with a negative charge (Figure 5). Phosphodiesterases are extracellularly expressed and could potentially cleave the adenosine-5′-monophopshate from the ADP-ribose of the ADP-ribosylated-HNP-1. These two latter examples of processing have not been described for HNP-1 so far. 

## 6. Future Perspectives of ADP-Ribosylation of Antimicrobials

The molecular and functional comprehension of the biological activities of AMPs and how these activities are regulated *in vivo* could represent a crucial step in understanding deeply how innate immunity is modulated, since AMPs are an important defence mechanism against invading microorganisms. The biochemical characterization of ADP-ribosylated antimicrobial peptides, including the interaction with both their molecular targets and biological membranes, is important to reach this goal. The study of the functional role of arginine-ADP-ribosylation of antimicrobial peptides could represent the starting point for finding new therapeutic opportunities. Both NMR and crystallography are by far the most potent tools in investigating the protein structure properties. Furthermore, improved structure-function prediction algorithms are another opportunity to develop synthetic peptide that mimics modified portions or inhibitors of this reaction for therapeutic use. Monitoring the ADP ribosylated peptide should be an interesting strategy to identify biological targets even though, some AMPs are not directly bactericidal, but exert their effect by immunomodulation [31]. Recent improved tools that include antibodies specific for ADP-ribosylarginine [32] or the possibility to generate site-specific ADP-ribosylated peptides [33, 34] will be useful to study and eventually treat pulmonary inflammation, other lung disorders as well as toxin-mediated diseases. The replacement with ornithine of the Arg linked with ADP-ribose could represent an alternative pathway for the regulation of the biological activities of HNP-1 through post-translational modification. It could be of interest to evaluate the effects of these modifications on HNP-1 functions. Since AMPs are rich in Arg but also contain amino acids potential substrate for phosphorylation (Table 1), it could be also of interest to evaluate whether the regulation of biological activities of antimicrobials by ADP-ribosylation or phosphorylation is restricted to HNP-1 or is a more generic mechanism.

## 7. Conclusions

In this review we have described how the ADP-ribosylation of specific Arg residues of HNP-1 affects its biological properties. The biological activities of HNP-1 are strictly dependent on the elevated cationicity due to the presence of Arg residues. Mono ADP-ribosylation is one example how post-translational modifications can affect the functions of peptides such as the *α*-defensin. In the era of microarray technology it has to be recognized that the analysis of the expression and regulation of single genes alone is not sufficient to understand the functional roles of proteins and peptides within normal or pathological situations. The interplay between ADP-ribosylation and phosphorylation, beyond the analysis of the functional effects that each single reaction can exerts on peptides belonging to the innate immunity system, should be also taken into consideration.

## Figures and Tables

**Table 1:** Human defensins.

*α*	DEF1_HUMAN neutrophil defensin 1	ACYC**R**IPACIAGE**RR**YGTCIYQG**R**LWAFCC
	DEF1_HUMAN neutrophil defensin 2	CYC**R**IPACIAGE**RR**YGTCIYQG**R**LWAFCC
	DEF3_HUMAN neutrophil defensin 3	DCYC**R**IPACIAGE**RR**YGTCIYQG**R**LWAFCC
	DEF4_HUMAN neutrophil defensin 4	VCSC**R**LVFC**RR**TEL**R**VGNCLIGGVSFTYCCT**R**VD
	DEF5_HUMAN defensin 5	ATCYC**R**TG**R**CAT**R**ESLSGVCEISG**R**LY**R**LCC**R**
	DEF6_HUMAN defensin 6	TCHC-**RR**SCYSTEYSYGTCTVMGINH**R**FCCL

*β*	DEFB1_HUMAN *β*-defensin 1	DHYNCVSSGGQCLYSACPIFTKIQGTCY**R**GKAKCCK
	DEFB2_HUMAN *β*-defensin 2	GIGDPVTCLKSGAICHPVFCP**RR**YKQIGTCGLPGTKCCKKP
	DEFB3_HUMAN *β*-defensin 3	GIINTLQKYYC**R**V**R**GG**R**CAVLSCLPKEEQIGKCST**R**GRKCC**RR**KK
	DEFB4_HUMAN *β*-defensin 4	ELD**R**ICGYGTA**R**C**R**KK-C**R**SQEY**R**IG**R**CPNTYA-CCL**R**K
